# Führen in der Krise – organisationales Krisenmanagement während der COVID-19-Pandemie („coronavirus disease 2019“) am Beispiel der Lebenshilfe Tirol

**DOI:** 10.1007/s11553-021-00914-0

**Published:** 2021-11-01

**Authors:** Priya-Lena Riedel, Vanesse Kulcar, Alexander Kreh, Martin Reiter, Barbara Juen

**Affiliations:** 1 Institut für Psychologie, Universität Innsbruck, Innrain 52f, 6020 Innsbruck, Österreich; 2 Lebenshilfe Tirol gem.Ges.mbH, Stadtgraben 7a, 6060 Hall in Tirol, Österreich

**Keywords:** Gesundheitspersonal, Organisationale Resilienz, Direktive Führung, Partizipation, Krisenkommunikation, Health care personnel, Organizational resilience, Directive leadership, Crisis communication, Participation

## Abstract

**Hintergrund:**

Die COVID-19-Pandemie („coronavirus disease 2019“) stellt im Gesundheitsbereich eine Herausforderung dar. Die Kenntnis organisationaler Schutz- und Risikofaktoren ist zentral zur Aufrechterhaltung des psychosozialen Wohlbefindens der Mitarbeitenden sowie der Versorgung von Klient:innen.

**Ziel:**

Das Ziel dieser qualitativen Untersuchung von Führungspersonal und Mitarbeiter:innen ist die Identifikation von spezifischen Schutz- und Belastungsfaktoren bei der Begleitung von Menschen mit Beeinträchtigung. Auf dieser Basis sollen Rückschlüsse auf notwendige Anpassungen der Führung in Krisensituationen möglich werden.

**Methodik:**

Zwischen Oktober und Dezember 2020 wurden online-basierte Expert:inneninterviews (*n* = 11) mit Mitarbeiter:innen der Lebenshilfe Tirol durchgeführt. Durch Anwendung der Grounded Theory wurde ein Modell zur Erklärung des Erlebens von Gesundheitspersonal sowie den Kontextfaktoren und Bewältigungsstrategien in der Behindertenbetreuung erstellt.

**Ergebnisse:**

Das Stresserleben während der COVID-19-Pandemie war durch Unsicherheit und Überforderung charakterisiert, welche durch die Neuartigkeit der Situation, einem Übermaß an Informationen sowie veränderte Arbeitsbedingungen unter verringerter Partizipation hervorgerufen wurden. Positives Erleben war mit Sinnerfülltheit sowie flachen Hierarchien und der Identifikation mit der Organisation assoziiert. Durch eine Anpassung des Führungsverhaltens, der durch flache Hierarchien gekennzeichneten Organisation, hin zu direktiven Entscheidungen konnte erfolgreich auf die Pandemie reagiert werden.

**Diskussion:**

Die Ergebnisse dieser Untersuchung weisen auf veränderte Anforderungen an das Führungsverhalten und einen Bedarf an verstärkter direktiver Führung während Krisen hin. Dabei erwiesen sich dezentrale Strukturen und ein laufender Dialog mit Mitarbeiter:innen, Klient:innen und Angehörigen als zentrale Resilienzfaktoren. Nur durch ausreichende Kommunikation und Inkludierung der Mitarbeiter:innen kann der Wechsel im Führungsstil akzeptiert werden.

## Abstract (en)

**Background:**

The COVID-19 (coronavirus disease 2019) pandemic poses a challenge to health care providers. Knowledge of organizational protective and risk factors is central to maintaining staff psychosocial well-being and client care.

**Objective:**

The aim of this qualitative study with caregivers and leadership personnel is to identify specific protective and stress factors of staff members accompanying people with disabilities. This allows conclusions to be drawn about necessary adjustments to leadership in crisis situations.

**Methodology:**

Between October and December 2020, online-based expert interviews (*N* = 11) were conducted with staff members of Lebenshilfe Tirol. By applying axial coding of grounded theory, a model was created to explain the experience of health care workers in disability care.

**Results:**

Stressful experience during the COVID-19 pandemic was characterized by uncertainty and a feeling of being overwhelmed, which were caused by the novelty of the situation, an excess of information, and altered working conditions with reduced participation. Positive experience was associated with meaningfulness as well as flat hierarchies and identification with the organization. By adapting the leadership behavior of the organisation, which before the crisis was characterized by flat hierarchies, it was possible to respond successfully to the pandemic.

**Discussion:**

The results of this study point to changed demands on leadership behavior and a need for increased directive leadership during crises. This change from participative to directive leadership has to be accompanied by dialogue with all stakeholders in order to be accepted.

## Hintergrund und Fragestellung

Die COVID-19-Pandemie („coronavirus disease 2019“) ist mit Beeinträchtigungen des psychischen Befindens assoziiert [26]. Besonders betroffen ist Personal im Sozial- und Gesundheitsbereich [13]. Zur Belastung tragen organisationale Stressoren bei, die in gesellschaftliche und politische Rahmenbedingungen eingebettet sind. Gleichzeitig können organisationale Strukturen auch als protektive Faktoren zur erfolgreichen Krisenbewältigung beitragen [16].

## Organisationale Stressoren

Als organisationale Stressoren werden stressverursachende oder verstärkende Faktoren der internen Strukturierung arbeitsgebender Organisationen bezeichnet. Die Pandemie kann insbesondere durch das geringe Maß an Kontrollierbarkeit in Kombination mit hohen Anforderungen negative Auswirkungen auf die psychische und physische Gesundheit der Mitarbeiter:innen haben [12, 23]. Im Zuge der COVID-19-Pandemie führte eine direktive Verteilung neuer Aufgaben zu einem Gefühl fehlender Wertschätzung [3]. Insbesondere in Organisationen mit geringem Maß an Unterstützung für Mitarbeiter:innen war das Risiko für Burnout und Arbeitsunzufriedenheit erhöht [1]. Arbeitszufriedenheit war dabei negativ mit psychischem Disstress assoziiert [7]. Auch in Bezug auf die Patient:innenversorgung zeigten sich negative Effekte [1]. Während der COVID-19-Krise stellten Unsicherheiten in Bezug auf die Informationsvermittlung und Handlungsstrategien Hauptstressoren dar, konkret ein gesteigertes Maß an Fachinformationen und neuartigen Hygieneregeln [6] bei gleichzeitig mangelndem pandemiebezogenem Wissen [8]. Zusätzliche Herausforderungen bestanden durch mangelnde Einarbeitung, Erfahrung und Unterstützung [6]. Desroches et al. (2021) berichteten von Konflikten zwischen Betreuungskräften und Administration, weil erstere nicht ausreichend in pandemiebezogene Planungs- und Entscheidungsprozesse involviert wurden [6]. Zudem waren Organisationen häufig unvorbereitet bezüglich der Maßnahmen zur Abschirmung von Risikogruppen [20]. Personalmangel sowie eine erhöhte Arbeitsbelastung des Personals mit direktem Patient:innenkontakt verschärften diese Probleme [3].

## Gesellschaftliche und politische Stressoren

Die organisationalen Stressoren sind eingebettet in gesellschaftliche und politische Strukturen, welche die Belastungen zusätzlich verschärfen können. So ist Pflegepersonal für Menschen mit Behinderung häufig in mehreren Einrichtungen tätig [6, 22], was insbesondere während der COVID-19-Krise als Stressor wirken kann [6]. Die Mehrfachbeschäftigung erhöht zudem das Übertragungsrisiko des Virus und stellt ein Hindernis bei der Logistik von Testungen dar. Zusätzliche Herausforderungen stellen veränderte Vorschriften für Schutzmaßnahmen durch die Politik [20] und ein rascher Wechsel an Handlungsempfehlungen dar [6]. Auch die fehlende gesellschaftliche Anerkennung von Mitarbeiter:innen in der sozialen Pflege wird als stressreich empfunden [8]. Dies spiegelt sich auch in der fehlenden finanziellen Unterstützung der Behindertenhilfe durch Krisenfonds wider [6].

## Protektive organisationale Wirkfaktoren

In Krisenzeiten sind Charakteristika des Arbeitsplatzes, die resilienzstärkend wirken können [9], besonders entscheidend. Zentral sind dabei Gefühle von Sicherheit, Ruhe, Verbundenheit, Selbst- und kollektiver Wirksamkeit, sowie positive Zukunftsorientierung [10, 26]. Dies kann mittels dezentraler Entscheidungsprozesse, Selbstadministration von Abteilungen, Weiterbildungsangeboten, sowie der Einbindung von Pflege ins Management gefördert werden [16]. Berufserfahrung und adäquates Training sowie Unterstützung zeigten sich bei Lancee et al. (2008) 2 Jahre nach Ausbruch der SARS-Pandemie („severe acute respiratory syndrome“) als stressreduzierende Faktoren [16]. Zentral ist während Gesundheitskrisen eine rasche, proaktive Kommunikation, welche die Sorge bei Mitarbeitenden reduzieren kann [7].

## Organisationale Resilienz

**Abb. 1:**
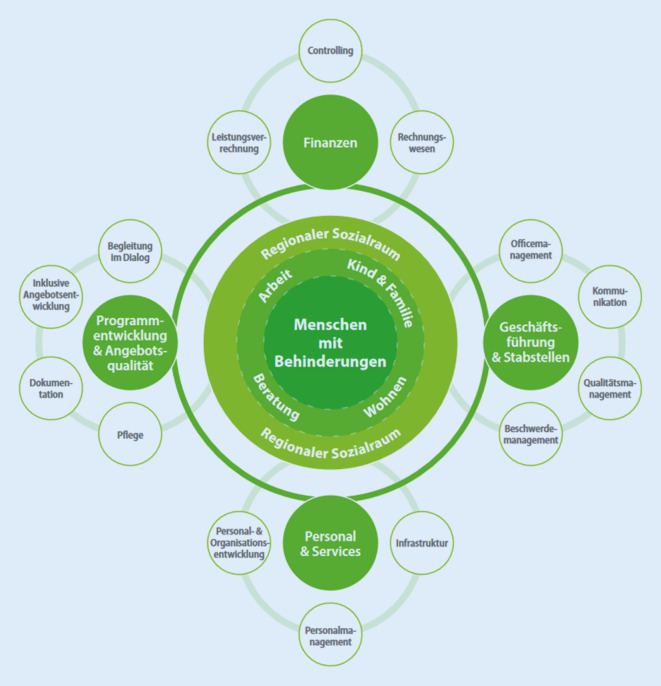
Organigramm der Lebenshilfe Tirol. (Quelle: [17], Copyright durch Lebenshilfe Tirol gem GmbH)

Organisationale Resilienz besteht aus der Widerstandsfähigkeit, der Erholungsfähigkeit und der Lernfähigkeit einer Organisation in Krisen [21]. In diesem Artikel sollen organisationale Resilienzfaktoren in der COVID-19-Pandemie am Beispiel einer Organisation für Menschen mit Beeinträchtigung analysiert werden. Bei der Lebenshilfe Tirol handelt es sich um eine Organisation, die Menschen mit Beeinträchtigungen in ihrer Lebensführung unterstützt. Die Unterstützung findet sowohl in Wohnheimen als auch in Arbeitseinrichtungen statt. Die Lebenshilfe zeichnet sich durch flache Hierarchien und breite Entscheidungskompetenzen aus (s. Abb. 1) und wurde daher mit Referenz auf Lancee et al. (2008) als Beispielorganisation für eine Untersuchung organisationaler Resilienzfaktoren ausgewählt [16]. Ziel ist dabei die Identifikation und weitere Vertiefung des Wissens über organisationale Resilienz und der Förderung der psychosozialen Gesundheit von Mitarbeiter:innen im Gesundheitsbereich während der COVID-19-Pandemie.

## Methode

Die vorliegende Studie erfasste mittels semistrukturierter Expert:inneninterviews qualitativ das Erleben von Mitarbeiter:innen und Führungskräften der Lebenshilfe Tirol während der ersten und der zweiten Welle der COVID-19-Pandemie. Zwischen 22.10.2020 und 14.12.2020 wurden online Leitfadeninterviews mit 11 Mitarbeiter:innen mit und ohne Führungsverantwortung durchgeführt (Dauer 39–79 min). Die Teilnehmenden wurden vor Beginn der Interviews bezüglich ihrer Rechte und der Pseudonymisierung aufgeklärt und stimmten dem Interview, dessen Aufnahme und dessen Nutzung schriftlich zu. Transkriptionen wurden erstellt und mit den Teilnehmenden zur Möglichkeit der Korrektur und Ergänzung diskutiert. Einer Veröffentlichung von organisationsbezogenen Daten wurde zugestimmt.

Im ersten Schritt der Analyse wurden die Inhalte der Interviews mittels zusammenfassender Inhaltsanalyse nach Mayring [19] schrittweise reduziert und zu Kategorien zusammengefasst. In der Folge wurden anhand des axialen Kodierens der Grounded Theory [24] Schlüsselkategorien gebildet. Dabei wurde eine Verbindung zwischen den Kategorien hergestellt und Hypothesen gebildet. Positive und negative Kontextfaktoren sowie Bewältigungsstrategien und Stresserleben wurden zueinander in Beziehung gesetzt. Auf diese Weise wurden die zentralen Resilienzfaktoren induktiv herausgearbeitet.

## Stichprobe

Sieben Teilnehmende waren männlich, vier weiblich. Das Alter lag zwischen 31 und 56 Jahren bei einer Berufserfahrung von einem bis 23 Jahren. Es waren 8 Mitarbeitende mit Führungsverantwortung und 3 ohne Führungsverantwortung aus verschiedenen Bereichen der Organisation vertreten.

## Ergebnisse

Im Folgenden werden die Hauptkategorien Stressoren (stresserzeugende und stressreduzierende) Kontextfaktoren und Strategien nach dem axialen Modell der Grounded Theory beschrieben.

### Stresserleben

Das subjektive Erleben der Interviewten war durch Sorge um die Klient:innen, Unsicherheiten, Stress und Überforderungsgefühle charakterisiert. Zu Beginn der Pandemie war die Sorge um die Familie groß und es bestand Angst bezüglich des Virus: „Das Anfangsgefühl war, jetzt kommt der Virus und dann bist du sofort tot“ (Interview 4).

Unsicherheit und Angst bezogen sich auch auf das Schutzmaterial und dessen Güte sowie auf mangelnde Trainings. Zudem wurde Ärger über ein Umfeld berichtet, welches sich nicht an Hygieneregeln hält, sowie Sorge über die Sicherstellung der Betreuung von Klient:innen bei Personalausfall durch Ansteckung. Außerdem herrschte Verunsicherung bezüglich möglicher ethischer und rechtlicher Grenzüberschreitungen.

Gleichzeitig war das Erleben der Mitarbeiter:innen gekennzeichnet durch Sinnerfülltheit und Selbstwirksamkeit bei der Arbeit. Das von der Organisation vor der Pandemie erarbeitete Selbstverständnis half den Mitarbeiter:innen, auch während der Krise das Gefühl der Sinnerfüllung nicht zu verlieren. Sowohl Mitarbeiter:innen als auch Klient:innen planten eigeninitiativ Projekte in den Zeiten der Krise, was aus Sicht einer Leitung „ein Ausdruck von einer Grundeinstellung von einer Lebensfreude von Bejahung auch in dieser Krisensituation [ihrer] Arbeit.“ (Interview 10) war.

Bei den Führungskräften erzeugte die erfolgreiche Bereitstellung von Schutzmaterial und Tests ein Gefühl der Selbsteffizienz. Auch die zunehmende Sicherheit im Umgang mit Verdachtsfällen erhöhte das Gefühl der Selbstwirksamkeit.

### Stresserzeugende Kontextfaktoren

Im Folgenden werden die stresserzeugenden Kontextfaktoren näher erläutert.

#### Neuartigkeit und sich rasch ändernde Information

Eine Herausforderung bezeichnete die *Neuartigkeit von Pandemiesituation und Maßnahmen*. Der rasche Umsetzungsversuch der Maßnahmen sorgte zu Beginn der Pandemie für Stress. Auch die häufige Veränderung von Vorgaben und eine Flut an sich *rasch verändernder oder auch fehlender Information* waren stressinduzierend und führten zunächst zu einer Handlungsunsicherheit bei den Mitarbeiter:innen. Für die lokalen Leitungen stellte das Filtern von relevanten Informationen und die Weiterleitung von akzeptierten Handlungsanweisungen eine Herausforderung dar. Schwierig fand diese „das Thema Dosierung und Frequenz von Kommunikation, weil es sich so schnell verändert. Genauso wann ist man zu früh dran und wann ist man zu spät dran“ (Interview 8).

#### Mangel an Vorbereitung auf ein pandemisches Geschehen und Fehlen von Handlungsplänen

Die Vorbereitung auf die Pandemiesituation wurde als nicht ausreichend empfunden. Fehlende Handlungspläne bedeuteten zu Beginn der Pandemie die spontane Schließung von Einrichtungen bei Infektionsfällen. Interviewte benannten ein *Fehlen von Krisenplänen* und einen Mangel an Schutzausrüstung. Nicht in allen Einrichtungen der Tagesbetreuung wurde sofort ein Training zum Anlegen der Schutzausrüstung für Quarantänestationen angeboten.

#### Mangelnde Sicherheit

Sorge um die *Güte des Schutzmaterials* wurden ebenfalls als Herausforderung genannt: „Die Güte [der Schutzanzüge] war glaube ich nicht unbedingt gut. Und trotzdem hat man in den Wohnhäusern, wo es Fälle gegeben hat, genau mit dem auch gearbeitet“ (Interview 9). Auch die Einführung von *Quarantänestationen* war mit Schwierigkeiten verbunden. Lokale Leitungen fürchteten eine Überforderung von Mitarbeiter:innen und es wurden Schwierigkeiten bei der Einhaltung von Quarantänemaßnahmen für Klient:innen berichtet.

#### Garantie der Versorgung der Menschen mit Behinderungen im Fall von massivem Dienstausfall

Interviewte antizipierten zu Beginn der Pandemie den *Ausfall der Betreuung* der Klient:innen bei eigener Ansteckung. Mitarbeiter:innen beschrieben ein Umfeld, welches sich nicht an Hygieneregeln hielt und so Ansteckung der Klient:innen oder der Mitarbeiter:innen möglich machte, was mit der Sorge assoziiert war „dass es dort zusammenbrechen kann, durch Mitarbeiter, die ausfallen oder ganze Mitarbeiterketten oder Diensträder“ (Interview 9). Als stressreich wurde auch die Unterbringung von Klient:innen bei Angehörigen beschrieben, welche selbst eine Risikogruppe darstellen. Während des Lockdowns belastete Mitarbeiter:innnen auch die Vereinsamung und der Mangel an Beschäftigung bei den Klient:innen: „Der Lockdown, den wir erlebt haben, ist um einiges kürzer als der, den die Menschen mit Beeinträchtigung erlebt haben, weil die gar nicht so schnell wieder zur Arbeit gehen durften. Die sind dann allein zuhause eingesperrt gewesen“ (Interview 5).

#### Rücknahme von Partizipation und Dialog am Beginn

Der Fokus lag zu Beginn der Pandemie bei der Aufrechterhaltung der Versorgung. Dialoge und Gespräche auf informeller Beziehungsebene wurden zurückgefahren. *Mangelnder Dialog* zwischen den Standortleitungen des Wohnens und der Arbeit zum Ablauf der Zuteilung der Mitarbeiter:innen aus dem Arbeitsbereich in den Wohnbereich führte zu Konflikten und Unzufriedenheit.

#### Veränderung der Arbeitsbedingungen auf allen Ebenen der Organisation

Die lokalen Leitungen hatten erhebliche *Mehrarbeit* durch tägliche Telefonate mit den Mitarbeiter:innen und Angehörigen sowie durch die Bewältigung von zusätzlichen Verwaltungsaufgaben: „Das war ein Wahnsinn fürs Zeitmanagement und auch der Mehraufwand, Maßnahmen weiterzuleiten, zu organisieren auch“ (Interview 6). Bei den Mitarbeiter:innen *verlängerte sich die Arbeitszeit* durch mobile Arbeit und durchgehende Verfügbarkeit für die Klient:innen, außerdem wurden Schwierigkeiten bei der Dokumentation berichtet. Herausforderungen bestanden zudem bei der *Vereinbarkeit von Homeschooling und Homeoffice*. Zugleich hatten auch die Führungskräfte mit Mehrfachbelastungen zu kämpfen, was besonders am Beginn dazu führte, dass es zu Missstimmungen zwischen den Ebenen kam.

#### Der Wechsel von Mitarbeiter:innen aus dem Arbeitsbereich in den Wohnbereich

Mitarbeiter:innen *wechselten von dem Arbeits- in den Wohnbereich*. Die veränderten Arbeitsbedingungen wurde von den lokalen Leitungen als stressreich beschrieben: „Die Dynamik in einem Wohnbereich und die in einem Arbeitsbereich ist eine ganz andere. Das ist grundsätzlich eine Herausforderung“ (Interview 9). Stress entstand durch den Mangel an Wissen und Übung in der neuartigen Arbeitsweise, veränderte Arbeitszeit und eine neuartige Arbeitsumgebung. Herausforderungen bestanden im Vertrauensaufbau zu den Klient:innen und innerhalb eines fremden Teams.

Die Herausforderungen konnten durch die in der Organisation herrschenden positiven Kontextbedingungen rasch abgefedert werden.

### Stressreduzierende Kontextfaktoren

Als besonders resilienzstärkend erwiesen sich die in der Organisation herrschenden flachen Hierarchien sowie die organisationale Identität. So wurde eine flexible Anpassung an die neue Situation erleichtert und die Arbeitsmotivation erhalten.

#### Flache Hierarchien

Die Organisation zeichnet sich durch *flache Hierarchien* aus, welche Entscheidungskompetenzen und Verantwortungsübernahme auf allen Hierarchieebenen garantieren. So wird nicht nur Führungspersonal sondern auch Mitarbeiter:innen ohne Führungsverantwortung in unterschiedliche Entscheidungen involviert: „[…] wir sagen nicht nur die Leitungen sollen entscheiden, sondern auch die Mitarbeiter in der direkten Zusammenarbeit mit dem Klienten. Dass die Mitarbeiter das Gefühl haben, sie dürfen, können, sollen entscheiden“ (Interview 1).

#### Identität der Organisation

Die *„Identität“ der Organisation* wird als gesellschaftlicher Einsatz für die Menschenrechte gesehen. Diese organisationale „Haltung“ trug die Mitarbeiter:innen und Leitungspersonen in der Krise. Sie stellten die Basis der organisationalen Resilienz und Krisenkompetenz sowie der Arbeitsmotivation dar. Auf allen Ebenen der Organisation wurde von Interviewpartner:innen über einen wertschätzenden Umgang unter allen Mitarbeiter:innen der Organisation, gegenüber Klient:innen und in Bezug auf das Verhältnis zu den Angehörigen berichtet. Es wird angestrebt, Ressourcen, Stressoren und Fähigkeiten der Mitarbeiter:innen zu erkennen und diese bei dem Umgang mit Herausforderungen zu unterstützen. Bei Verordnungen und Vereinbarungen besteht Verbindlichkeit. Außerhalb der Verordnungen sollen Mitarbeiter:innen Verantwortung für ihre Entscheidungen und Handeln innerhalb der Organisation übernehmen. Entscheidungen sollen nachvollziehbar auf der Basis der organisationalen Identität getroffen werden. In der Organisation wird eine klare Kommunikation propagiert. Nach anfänglichen Schwierigkeiten wurde diese Form der Kommunikation auch in der Krise rasch wieder erreicht.

### Strategien der Krisenbewältigung

Im Folgenden werden wir die Strategien der Führungskräfte beschreiben, die dazu führten, dass sich das System rasch anpassen konnte. Krisenbewältigung bedeutete für die Führungskräfte zunächst vor allem Schutz und gute Kommunikation.

#### Rascher Schutz der Mitarbeiter:innen und Klient:innen

Zum Schutz der *Mitarbeiter:innen und Klient:innen* wurde zu Beginn des Pandemiegeschehens auf Improvisation bei der Beschaffung von Schutzmaterial gesetzt. Mit dem Ende der Knappheit wurden Konzepte für Notfalllager erarbeitet. Die Verfügbarkeit von Schutzausrüstung und klaren Handlungsanweisungen konnte das Sicherheitsgefühl der Mitarbeiter:innen nach einer anfänglichen Unsicherheitsphase wiederherstellen. Die Durchführung von präventiven Testungen des Personals in Wohneinrichtungen wurde durch die private Beschaffung von Testmaterial möglich. Auch das erhöhte das Gefühl der Sicherheit.

#### Zunehmende Sicherheit im Umgang mit Verdachtsfällen

Durch die fortgesetzte Betreuung war die individuelle Bezugnahme auf die Bedürfnisse der Klient:innen und Angehörigen möglich. Im Verlauf der Krise wurden *klare Regeln zum Umgang mit Verdachtsfällen* etabliert, welche das Sicherheitsgefühl erhöhten und Schließungen von Arbeitseinrichtungen verhinderten: „Wenn jetzt ein Verdachtsfall in einer Einrichtung ist, dann reden wir nicht von Schließung, sondern von einer Abklärung des Verdachtsfalles“ (Interview 10).

#### Einrichtung von Quarantänestationen

Im Verlauf der ersten Infektionswelle wurde die *Einrichtung von organisationseigenen Quarantäneeinrichtungen* initialisiert. Hintergrund sind die Schwierigkeit der Weiterbegleitung von Klient:innen in einem Krankenhaussetting, wie auch die Vulnerabilität der Angehörigen als Risikogruppe. Ziel war hierbei die Sicherstellung der Versorgung erkrankter Klient:innen sowie der Schutz der Angehörigen vor Ansteckung. Handlungsanweisungen zum Betreiben einer Quarantäneeinrichtung wurden zur Verfügung gestellt.

#### Klare verbindliche Anweisungen durch die Geschäftsleitung

Die Geschäftsführung spielte die zentrale Rolle und konnte durch *klare und verbindliche Dienstanweisungen* den Mitarbeiter:innen Verantwortungsübernahme und Sicherheit garantieren. Das direktive Entscheidungs- und Führungsverhalten konnte eine rasche und situationsangepasste Umsetzung von Maßnahmen ermöglichen. Dienstvorschriften mit Handlungsanweisungen waren für alle Mitarbeiter:innen und Führungspersonen verbindlich, was vom Leitungspersonal als hilfreich in der Kommunikation mit Mitarbeiter:innen empfunden wurde: „Das war für mich als Leitungsperson sehr hilfreich. Zu sagen vor den Mitarbeitern: Die Geschäftsführung hat das entschieden, wir brauchen da nicht mehr zu diskutieren, sondern das ist eine Vorgabe, Dienstanweisung“ (Interview 7).

#### Krisenteam

**Abb. 2:**
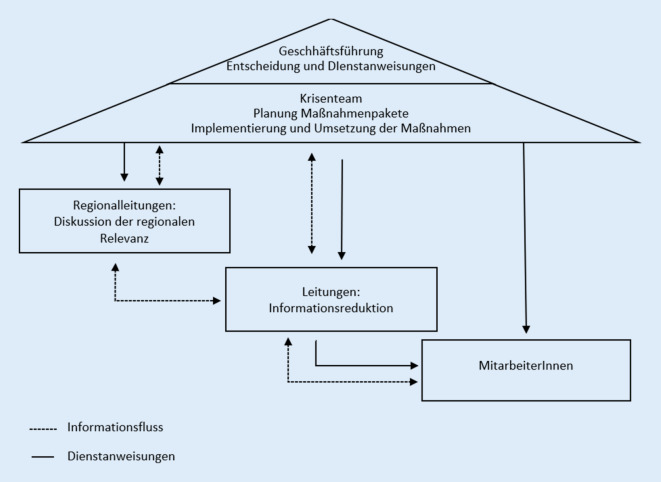
Entscheidungsmanagement und Informationsfluss in der Krise

Ein *Krisenteam* diente als Beratungsgremium für die Geschäftsführung. Das Expertengremium veränderte, modifizierte und kommunizierte bestehende Regelungen in Zusammenarbeit mit der Geschäftsführung. Das Gremium trat in konstanter und interdisziplinärer Besetzung auf. Die Zuständigkeit dieses Expertengremiums lag in der Aufbereitung von Informationsinhalten sowie der zentralen Steuerung und Überwachung von Informations- und Kommunikationsabläufen (s. Abb. 2). Auf der Basis aktueller Informationen wurden Pläne für den pflegerischen Bereich, Begleitbereich, Besuchs‑, Angehörigenbereich und das Vorgehen bei Öffnungen entwickelt. Spezifische Fragestellungen wurden durch thematische Untergruppen erarbeitet. Die Inhalte bezogen sich auf Kurzarbeit, Freistellungen, interne und externe Kommunikation, die Übersetzung der gesetzlichen Richtlinien in eine nutzer:innenfreundliche Sprache sowie Entscheidungen über die Umsetzung der gesetzlichen Verordnungen innerhalb der Organisation.

#### Frühe und stetige Mitarbeiter:innenkommunikation

Eine *frühe und stetige Kommunikation* sorgte für die Informiertheit aller Mitarbeiter:innen der Organisation. Unter den Bedingungen von ständigen Veränderungen von Gesetzmäßigkeiten, Regelungen sowie dem dynamischen Infektionsgeschehen waren angepasste Informationsfrequenz und Dichte essenziell. Diese fand laut Interviewpartner:innen auch mithilfe des Ausbaus digitaler Angebote statt. Es wurden Videokonferenzen, E‑Mails, Handlungsanweisungen im Intranet und ein FAQ-Katalog zum Thema COVID-19 zur Verfügung. Zudem bestanden Angebote für Schulungen in dem Bereich IT, die den Mitarbeiter:innen leichtere Nutzung von Onlinemedien für die Betreuung der Klient:innen ermöglichten. Dabei ist es „nicht immer nur um das Berufliche gegangen, sondern auch um den Menschen, wie es der Person geht“ (Interview 8).

#### Kommunikation und Einbindung von Klient:innen und Angehörigen

Seit Beginn der Pandemie fand aktive *Kommunikation und Einbindung von Klient:innen und Angehörigen* statt. Im Verlauf konnten die Menschen mit Behinderungen durch Onlinekonferenzen mit der Geschäftsführung aktiv ihre Bedürfnisse im Hinblick auf die Pandemie benennen.

#### Nutzung der lokalen Leitungsebene als Schnittstelle

Die* lokalen Leitungen dienten als Schnittstellen* und waren im Verlauf der Pandemie sowohl mit der zweiten Führungsebene als auch mit den Mitarbeiter:innen vor Ort sowie Angehörigen vernetzt. Unter den Bedingungen von raschen Veränderungen von Gesetzmäßigkeiten Regelungen sowie dem dynamischen Infektionsgeschehen wurden Informationsfrequenz und Dichte angepasst. Für Mitarbeiter:innen und Angehörige waren lokale Leitungen der zentrale Ansprechpartner. In Fällen von herausfordernden Situationen für einzelne Mitarbeiter:innen, Betreuungsverpflichtungen, Risikogruppen, Überforderungen waren die Leitungen vor Ort für die Mitarbeiter:innen telefonisch oder digital verfügbar. Dabei wurden auch individuelle Lösungen erarbeitet.

#### Wertschätzung durch Anerkennung der Behindertenpflege als systemrelevant

Die Organisation initialisierte die *Anerkennung der Behindertenhilfe als systemrelevant*, Prämienzahlungen, Zulagen und Anreize bei Testungen. Durch diese Maßnahmen wurden finanzielle und berufliche Sicherheit sowie Wertschätzung vermittelt. Bedürfnissen der Mitarbeiter:innen wurde individuell begegnet, wie in Interview 4 beschrieben: „[…] die Sonderfreistellung, die ist mehr als die Regierung gibt. […]. Sei es für Mitarbeiter einer Risikogruppe oder bei persönlichen Betreuungspflichten für Kinder, dass man nicht arbeiten kann. Die Freistellungsmöglichkeit, die hat die Lebenshilfe dann ausgeweitet. Nun gibt es ganz viele individuelle Möglichkeiten für die Mitarbeiter, dass sie keine Angst haben brauchen“. Zudem gab es Unterstützungssysteme zwischen den Mitarbeiter:innen. Zur Psychohygiene wurden Intervisionen und Supervisionen für Mitarbeiter:innen sowie Coachings für Leitungspersonen weitergeführt. Auch wurde die Hinzuziehung von bezahlter psychologischer oder psychotherapeutischer Beratung angeboten.

### Gewonnene Hypothesen

Aus den Daten konnten folgende Hypothesen generiert werden:

1. Gute Krisenkommunikation und Schutz der Mitarbeiter:innen und Klient:innen sind zentrale Wirkfaktoren.
2. Der krisenbedingt notwendige Wechsel zu hierarchischer Führung bedingt anfängliche Konflikte.
3. Die Erholung des Systems wird beschleunigt durch vor der Pandemie angelegte flache Hierarchien und organisationale Identität.
4. Laufender Dialog mit allen Stakeholdern, wertschätzender Umgang und Fokus auf organisationale Gerechtigkeit sind Hauptresilienzfaktoren.

## Diskussion

Bei den Interviewpartner:innen der dargestellten Organisation war die Akzeptanz für einen veränderten Führungsstil hoch, da sie auf Transparenz, Vertrauen und Sinnerfülltheit bei der Arbeit sowie der Identifikation mit den Werten der Organisation aufbaute. Die vor der Krise etablierten flachen Hierarchien hatten das Vertrauen der Mitarbeiter:innen in die lokale Leitung als zentrale Ansprechpersonen gefördert, worauf auch in der Krise trotz des direktiveren Führungsstils zurückgegriffen werden konnte. Der stressreduzierende Effekt durch die Organisation [28] sowie die Bedeutung von Verständnis und Kommunikation durch das Leitungspersonal für die psychische Gesundheit [18] werden auch in anderen Untersuchungen betont. Dabei kommt es besonders auf organisationale Gerechtigkeit an, die sich als Beziehungsgerechtigkeit, einem Interesse der Führungskraft am einzelnen Mitarbeitenden, sowie Entscheidungsgerechtigkeit, dem Erleben von transparenten und gerechten Entscheidungen, äußert [16]. In vorhergehenden Untersuchungen wurde die Verbundenheit mit der Organisation als eine zentrale Basis für formelle und informelle Unterstützung während einer Krise betont [16].

Im Zuge der Pandemiebewältigung erwies sich vor allem am Beginn der rasche Wechsel zu einem hierarchischen Führungsverhalten als notwendig. Die Vermittlung von Sicherheit, der Informationsfluss sowie die formalisierte Unterstützung von Mitarbeiter:innen und Klient:innen durch das Unternehmen wären anders nicht zeitnah durchsetzbar gewesen. Die zentrale Organisation ermöglichte die Beschaffung von Schutzmaterial, die Einrichtung von Quarantänestationen sowie die Garantie einer raschen und verlässlichen Umsetzung aller Sicherheitsmaßnahmen. Der Herausforderung der Krisenkommunikation wurde mit einem zentralen und speziell eingerichteten Krisenteam begegnet. Die gebündelte und schnelle Verteilung von Handlungsanweisungen schaffte ein Gefühl der Handlungssicherheit. Die Ergebnisse bestätigen frühere Befunde, wo klare und schnell kommunizierte Informationen Angst- und Unsicherheitsreaktionen der Mitarbeiter:innen während Krisen reduzieren [7, 11, 18]. Wesentlich war es allerdings, vor diesem Hintergrund neue Strukturen des Dialogs und des Austausches mit allen Stakeholdern zu installieren. Auch das ist in der Literatur gut belegt [16]. Förderlich war zudem die offene Kommunikation des Stresserlebens unter den Mitarbeiter:innen. Auch dies bestätigt vorhergehende Untersuchungen, bei denen soziale Unterstützung zu einer Reduktion von Angst und Stress beiträgt [27]. Diese kann eine positive Einstellung und korrigierende oder kontextualisierende Erfahrungen vermitteln [4, 14] und das Gefühl der Selbstwirksamkeit verstärken [27].

Die Pandemie hat von allen Mitarbeiter:innen im Sozial- und Gesundheitswesen viel verlangt. Besondere Wertschätzung und Dank sind daher wesentliche Unterstützungsfaktoren. Formalisierte Unterstützung in Form von Supervisionen, die auch vor der Krise angeboten wurden, wurden in Rahmen der Pandemie durch das das Angebot von Psychotherapie erweitert. Dieses Vorgehen steht im Einklang mit der Empfehlung von Banks et al. (2020) sowie Walton et al. (2020; [2, 25]). Auch fand innerhalb der Organisation eine Absicherung der Mitarbeiter:innen durch finanzielle Unterstützung in Form von Prämienzahlungen statt. Derartige finanzielle Anerkennung ist auch in anderen Untersuchungen mit einer verbesserten psychischen Gesundheit der Mitarbeiter:innen verbunden [28]. Die Lebenshilfe konnte zudem die Anerkennung der Behindertenpflege als systemrelevant erzielen und so die gesellschaftliche Bedeutsamkeit hervorheben und die Aufrechterhaltung der Versorgung garantieren. Die Relevanz derartiger Maßnahmen wird auch von Kavanagh et al. [15] betont.

## Limitationen

Die geringe Interviewanzahl in der vorliegenden Untersuchung limitiert die Verallgemeinerbarkeit der Ergebnisse. Die qualitative Methode erlaubt jedoch einen tieferen Einblick in das Erleben der Interviewten, aus denen Hypothesen abgeleitet werden können, die durch die Literatur unterstützt werden. Da sich die Lebenshilfe durch eine besondere organisationale Struktur auszeichnet, eignet sich diese Organisation daher als Best-practice-Beispiel. Wir gehen davon aus, dass die dargestellten Erkenntnisse auf andere Kontexte übertragbar sind und entsprechend quantitativ überprüft werden können. Im Rahmen des derzeit laufenden Projekts No-Fear [5] wird in einer Mixed-method-Studie das Gesundheitspersonal in der COVID-19-Pandemie („coronavirus disease 2019“) von uns untersucht und wesentliche Schutz- und Risikofaktoren herausgearbeitet. Die vorliegende Studie stellt einen Baustein in dem größer angelegten Projekt dar.

## Fazit für die Praxis

- Schutz- und Risikofaktoren aus dem Bereich der sozialen Pflege können bestätigt werden.
- Schutzfaktoren können gute Krisenkommunikation, ein Fokus auf den Schutz der Mitarbeiter:innen und Klient:innen, zugleich jedoch flexible anpassungsfähige Strukturen, die es ermöglichen, die psychosozialen Folgeschäden der Maßnahmen einzugrenzen, darstellen.
- Präventiv helfen flache Hierarchien und ein klares, an Wertschätzung und ein klares, an Respekt vor Grundrechten orientiertes Leitbild.
